# Visualizing Immunization Registry Data to Identify Places With Low Rates of HPV Vaccination Initiation in a Rural State

**DOI:** 10.5888/pcd17.190350

**Published:** 2020-03-05

**Authors:** Natoshia M. Askelson, Seungwon Kim, Youn Soo Jung, Emily E. Adam, Grace Ryan, Nicole L. Novak, Bethany Kintigh, Don Callaghan, Margaret Carrel

**Affiliations:** 1 Department of Community and Behavioral Health, University of Iowa, Iowa City, Iowa; 2 Public Policy Center, University of Iowa, Iowa City, Iowa; 3 Department of Geographical and Sustainability Sciences, University of Iowa, Iowa City, Iowa; 4 Bureau of Immunization and Tuberculosis, Iowa Department of Public Health, Des Moines, Iowa

**Figure:**
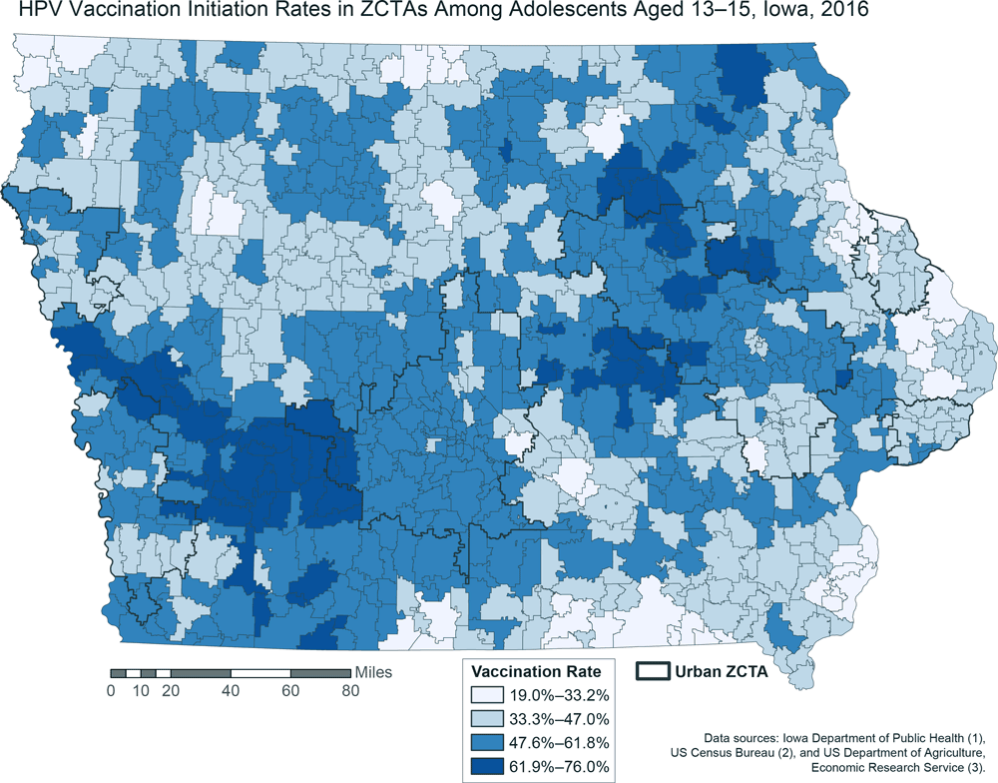
Covariate-adjusted initiation rates for human papillomavirus vaccination in zip code tabulation areas (ZCTAs) among adolescents aged 13 to 15, Iowa, 2016. Rates were spatially smoothed to account for small populations. The state-level mean vaccination rate for all ZCTAs was 47.9% (range, 0%–100%; standard deviation, 16.2%). All ZCTAs have initiation rates below the *Healthy People 2020* target of 80% completion. Urban ZCTAs are indicated in black outlines. Vaccination initiation rates exhibit no clear association with location. Data sources: Iowa Department of Public Health (1), US Census Bureau (2), and US Department of Agriculture, Economic Research Service (3). Abbreviation: ZCTA, zip code tabulation area.

## Background

Vaccination against human papillomavirus (HPV) protects against cancer and other diseases associated with the virus. Currently, the Advisory Committee on Immunization Practices recommends that boys and girls aged 11 and 12 receive a 2-dose vaccination series. Despite this recommendation, uptake of the HPV vaccine is low in comparison with uptake of other vaccines given at the same age (4). Some studies using national survey data identified rural residence as a risk factor for delayed HPV vaccination or no vaccination (5). However, national surveys often do not include enough rural children to adequately examine this risk factor. In this study, we used data from Iowa’s robust state immunization registry to examine the relationship between location and HPV vaccination initiation.

## Data Sources and Map Logistics

We used ecological data on the number of children in each zip code born in years 2001, 2002, and 2003 and the number that received HPV vaccinations as provided by the Iowa Department of Public Health via the Iowa Immunization Registry Information System (IRIS) for 2016. We selected adolescents aged 13 to 15 to capture data on those who should have completed the 2-dose vaccination series. IRIS tracks vaccines administered to residents of Iowa, and in 2016 the system accounted for 98% of the state’s population (1).

To examine the association between location and HPV vaccination initiation, we used regression modeling and controlled for the following variables at the level of zip code tabulation area (ZCTA) (Table 1): insurance coverage (percentage of population with public insurance and percentage with private insurance), race/ethnicity (percentage of population that is non-Hispanic white), poverty (percentage of population with income below 150% of the federal poverty level), education (percentage of population with a bachelor’s degree and percentage with a high school diploma) (2). The variables were entered into the model as proportions.We also included information on whether the ZCTA was designated by the Health Resources and Services Administration as a Health Professional Shortage Area (6), whether the ZCTA crossed the border into another state, and whether the ZCTA was identified as a “new destination” community (defined as a community that had experienced recent growth in the Hispanic population) (7). We determined rural status by using rural–urban commuting area codes (3). ZCTAs with rural–urban commuting area codes 1, 2, or 3 were considered urban, and all others were considered rural. The outcome variable was the count of children aged 13 to 15 who received the first HPV vaccine among the total number of children in each ZCTA. We used binomial regression with a conditional autoregressive prior and a Gaussian noise term to capture the spatially correlated random effects and the unstructured overdispersion in the model (8). The posterior distribution of parameters was generated by Markov Chain Monte Carlo procedures in OpenBUGS using R2OpenBUGS package in R (9). Posterior means of the vaccination rates in each ZCTA were inferred to generate a spatially smoothed map. The population-level covariates were used for smoothing to adjust for high variance and low stability of estimates in ZCTAs with small population sizes. The posterior means of the vaccination rate ratios were calculated in R and then imported into Esri’s ArcMap version 10.7 and mapped according to equal intervals by using a continuous color scheme. Urban ZCTAs, as defined by rural–urban commuting area codes, were also mapped to demonstrate urban and rural vaccination initiation rates.

**Table 1:** Descriptive Statistics for ZCTAs in Iowa (N = 933) for Variables Included In Regression Model, 2016

Variable	No. (%)	Mean (SD), %
Designated as a Health Professional Shortage Area^a^	432 (46.3)	—
Crosses into a bordering state	123 (13.2)	—
Designated as a “new destination” community^b^	52 (5.6)	—
Designated as rural^c^	376 (40.3)	—
Percentage of population with public insurance	—	32 (19)
Percentage of population with private insurance	—	70 (21)
Percentage of non-Hispanic white persons	—	96 (7)
Percentage of population with income below 150% federal poverty level	—	9 (6)
Percentage of population with bachelor’s degree	—	19 (10)
Percentage of population with high school diploma	—	92 (7)

Abbreviation: —, not applicable; ZCTA, zip code tabulation area.

a A geographic area with a shortage of primary care, dental, or mental health providers and services (6).

b Defined as a community that had experienced recent growth in the Hispanic population (7).

c Determined by using rural–urban commuting area codes (3). ZCTAs with rural–urban commuting area codes 1, 2, or 3 were considered urban, and all others were considered rural.

## Highlights

We found significant geographic variation in HPV vaccination initiation, with both high and low initiation rates in both urban and rural ZCTAs. When we controlled for zip code–level variables, initiation rates between urban zip codes and rural zip codes were not significantly different (rate ratio, 1.02; 95% credible interval, 0.95–1.10) (Table 2). The proportion of the population with a bachelor’s degree was negatively associated with vaccination initiation (rate ratio, 0.45; 95% credible interval, 0.30–0.67). The following characteristics were positively associated with vaccination initiation: proportion of the population with public insurance (rate ratio, 1.47; 95% credible interval, 1.08–2.04), proportion of the population with private insurance (rate ratio, 1.61; 95% credible interval, 1.17–2.21), and proportion of the population with a high school diploma (rate ratio, 2.90; 95% credible interval, 1.48–6.73). We found no other significant associations with vaccination initiation.

**Table 2:** Results From Regression Model for HPV Vaccination initiation Among Adolescents Aged 13 to 15, Iowa, 2016

Variable	Rate Ratio^a^ (95% Credible Interval)
Designated as a Health Professional Shortage Area^b^	1.00 (0.93–1.09)
Crosses into a bordering state	0.94 (0.84–1.04)
Designated as a “new destination” community^c^	1.09 (0.96–1.24)
Designated as rural^d^	1.02 (0.95–1.10)
Proportion of population in ZCTA with public insurance	1.47 (1.08–2.04)
Proportion of population in ZCTA with private insurance	1.61 (1.17–2.21)
Proportion of non-Hispanic white persons in ZCTA	0.71 (0.39–1.23)
Proportion of population in ZCTA with income below 150% of federal poverty level	0.84 (0.42–1.70)
Proportion of population in ZCTA with bachelor’s degree	0.45 (0.30–0.67)
Proportion of population in ZCTA with high school diploma	2.90 (1.48–6.73)

Abbreviation: ZCTA, zip code tabulation area.

a The posterior mean of the rate ratio.

b A geographic area with a shortage of primary care, dental, or mental health providers and services (6).

c Defined as a community that had experienced recent growth in the Hispanic population (7).

d Determined by using rural–urban commuting area codes (3). ZCTAs with rural–urban commuting area codes 1, 2, or 3 were considered urban, and all others were considered rural.

## Action

Immunization registries can be important data sources for public health practitioners to identify priorities for immunization interventions in targeted locations and subpopulations. We found significant geographic variation in HPV vaccination initiation rates across Iowa. However, when we controlled for zip code–level variables, rurality was not significantly associated with HPV vaccination initiation at the ZTCA level. Registry data provide more nuanced insight than national survey data into which geographic areas are at risk for undervaccination and could be potential intervention targets.

Visualizing vaccination data as we did has several implications for groups invested in improving vaccination rates. First, our map can help multiple stakeholders, including local and state health departments, insurers, and nonprofit organizations, determine where to focus financial and physical resources to improve vaccination initiation rates. Moreover, previous research in Iowa identified gaps in data related to vaccine delivery as a challenge for clinics and health care systems (10). Zip code–level analysis could help clinics and local health departments to better understand the population they serve and focus their vaccine delivery efforts. Future research should examine other predictors of spatial clustering of vaccination initiation rates beyond rural–urban location. Finally, our results show that state health departments can leverage relationships with university partners (or others with the capacity to perform complex geographic analyses) to better use available immunization data. Through this collaborative project, we provided our partners with an actionable map that will inform their outreach efforts and their work to improve HPV vaccination rates in Iowa.
